# TiO_2_ Nanomembranes Fabricated by Atomic Layer Deposition for Supercapacitor Electrode with Enhanced Capacitance

**DOI:** 10.1186/s11671-019-2912-3

**Published:** 2019-03-13

**Authors:** Farah Naeem, Sumayyah Naeem, Yuting Zhao, Dingrun Wang, Jing Zhang, YongFeng Mei, Gaoshan Huang

**Affiliations:** 1 Department of Materials Science, Fudan University, 220 Handan Road, Shanghai, 200433 People’s Republic of China; 2 State Key Laboratory for Modification of Chemical Fibers and Polymer Material Science and Engineering, Donghua University, Shanghai, 201620 People’s Republic of China; 3 College of Science, Donghua University, Shanghai, 201620 People’s Republic of China

**Keywords:** Atomic layer deposition, TiO_2_ nanomembranes, Electrode, Supercapacitor

## Abstract

TiO_2_ is a promising environment friendly, low cost, and high electrochemical performance material. However, impediments like high internal ion resistance and low electrical conductivity restrict its applications as electrode for supercapacitor. In the present work, atomic layer deposition was used to fabricate TiO_2_ nanomembranes (NMs) with accurately controlled thicknesses. The TiO_2_ NMs were then used as electrodes for high-performance pseudocapacitors. Experimental results demonstrated that the TiO_2_ NM with 100 ALD cycles had the highest capacitance of 2332 F/g at 1 A/g with energy density of 81 Wh/kg. The enhanced performance was ascribed to the large surface area and the interconnectivity in the case of ultra-thin and flexible NMs. Increased ALD cycles led to stiffer NMs and decreased capacitance. Moreover, one series of two supercapacitors can light up one light-emitting diode with a working voltage of ~ 1.5 V, sufficiently describing its application values.

**Electronic supplementary material:**

The online version of this article (10.1186/s11671-019-2912-3) contains supplementary material, which is available to authorized users.

## Introduction

With the maturation of energy storage technology [1], supercapacitors have received vast attention due to their high power density, fast charge-discharge rate, and good cycling performance [2–4]. Pseudocapacitor is an important class of supercapacitors, which can deliver attractive high capacitance and energy density compared with electrochemical supercapacitors [5–7]. In the past few decades, the transition metal oxides (e.g., RuO_2_ [8], MoO_2_ [9], MnO_2_ [10], Ni/NiO [11], Co_3_O_4_ [12], and TiO_2_ [13]) and hydroxides [14–16] were used as classic electrode materials for pseudocapacitors owing to low cost, low toxicity, multiple oxidation states [17], and great flexibility in structures and morphology. However, their thermal instability, impurity defects, and rate capability are usually limited by the inadequate conductivity to support fast electron transport required by high rates. In order to solve these problems, low-dimensional TiO_2_ structures (1D, 2D, 2D + 1D, and 3D) with high surface-to-volume ratio, good surface structure, great electrical and thermal stability, favorable energy band gap properties, and high dielectric constant have been engaged as promising electrode materials for supercapacitors [18–22]. Especially, we think that 2D nanomembrane (NM) structures with excellent flexibility should have great potential in electrode applications. The thickness control of nanomembrane is therefore crucial in fabricating functional devices in well-defined nanoworld [23]. In addition, large-scale manufacturing of nanoscale materials is also crucial for practical applications [24]. One may note that atomic layer deposition (ALD) is a captivating technique used to construct nanodevices [25, 26]. This powerful technique can deposit thin films layer by layer with accurate thickness control and can conformally cover 3D structures with high aspect ratio [27–30], and the productively can thus be greatly enhanced. In the current work, we present the fabrication of 2D TiO_2_ NMs with different thicknesses by performing ALD on 3D porous polymer template with large surface area [31, 32]. Microstructural characterization elucidates that the crystal structure of NM is a mixture of anatase and rutile phases. Electrochemical characterizations demonstrate that the ultra-thin and flexible NMs have the enhanced performance due to the large surface area and the interconnectivity among the NMs. The improved ion transportation causes Faradaic reaction on the surface as well as in the bulk [33], resulting in increased capacitance and energy densities.

## Methods

### Fabrication of TiO_2_ NMs

TiO_2_ NMs with various thicknesses (100, 200, and 400 ALD cycles) were deposited on a commercially available polyurethane sponge by using ALD technique. Tetrakis dimethylamide titanium (TDMAT) and de-ionized (DI) water were used as precursors in the presence of nitrogen (N_2_) gas which served as both carrier and purge gases. The flow rate of the carrier gas was 20 sccm. A typical ALD sequence includes TDMAT pulse (200 ms), N_2_ purge (20,000 ms), H_2_O pulse (20 ms), and N_2_ purge (30,000 ms). The precursors used were purchased from J&K Scientific Ltd., China. The precursor conformally covered the three-dimensionally porous sponge, which led to promoted productivity due to the large surface area of the template [34]. The TiO_2_-coated sponges were calcinated at 500 °C for 4 h in an O_2_ flow of 400 mL/min, and the template was completely removed. The resultant TiO_2_ NMs were crushed and cleaned in ethanol, hydrochloric acid (HCl), and DI water.

### Preparation of Electrode

In order to fabricate high-performance supercapacitor, TiO_2_ NMs with 100, 200, and 400 ALD cycles were used as the active material and polytetrafluoroethylene (PTFE) was used as binder. The contents of TiO_2_ NMs and binder were 90 wt% and 10 wt%, respectively. A homogeneous TiO_2_ NMs slurry was obtained by mixing the NMs and binder with a small quantity of ethanol, and a milling process was engaged. The prepared uniform slurry was deposited onto the cleaned nickel foam and then the sample was degassed at 60 °C for 2 h in vacuum. In order to complete the electrode fabrication, the sample was pressed under 10 MPa pressure. The prepared TiO_2_ NMs electrode was soaked in 1 M KOH solution for 12 h to activate the electrode. The loading densities of active materials were about ~ 1.5 mg cm^−2^ for all electrodes. The mass of the TiO_2_ NMs on nickel foam was obtained by calculating the mass difference between the electrode and nickel foam [35].

### Microstructural Characterization

The crystallographic structure of the TiO_2_ NMs was inspected by X-ray diffraction technique (XRD). The XRD patterns were recorded by using a Bruker D8A Advanced XRD with Cu Kα radiation (*λ* = 1.5405 Å). The morphology of TiO_2_ NMs was examined by scanning electron microscopy (SEM, Zeiss Sigma). The Raman spectra of the samples were carried out on a Horiba Scientific Raman spectrometer (*λ* = 514 nm). The elemental analysis and chemical state of the TiO_2_ NMs were obtained by using a PHI 5000C EACA X-ray photoelectron spectroscope (XPS), with C 1s peak at 284.6 eV as the standard signal. Atomic force microscopy (AFM, Dimension Edge, Bruker, USA) with tapping mode was used for surface topography of TiO_2_ NMs.

### Electrochemical Characterization

Three**-**electrode system was utilized to study the electrochemical properties of the TiO_2_ NMs working electrode where Ag/AgCl and platinum foil were acted as a reference electrode and counter electrode, respectively. The cyclic voltammetry (CV), chronopotentiometry (CP), and electrochemical impedance spectroscopy (EIS) measurements were accomplished on a Chenhua CHI 660E electrochemical workstation at 25 °C in 1 M KOH aqueous solution. EIS results were obtained over the frequency range of 100 KHz to 1 Hz with an amplitude of 5 mV. The calculation methods of specific capacitances and energy/power densities are described in Additional file 1.

## Results and Discussion

**Fig. 1:**
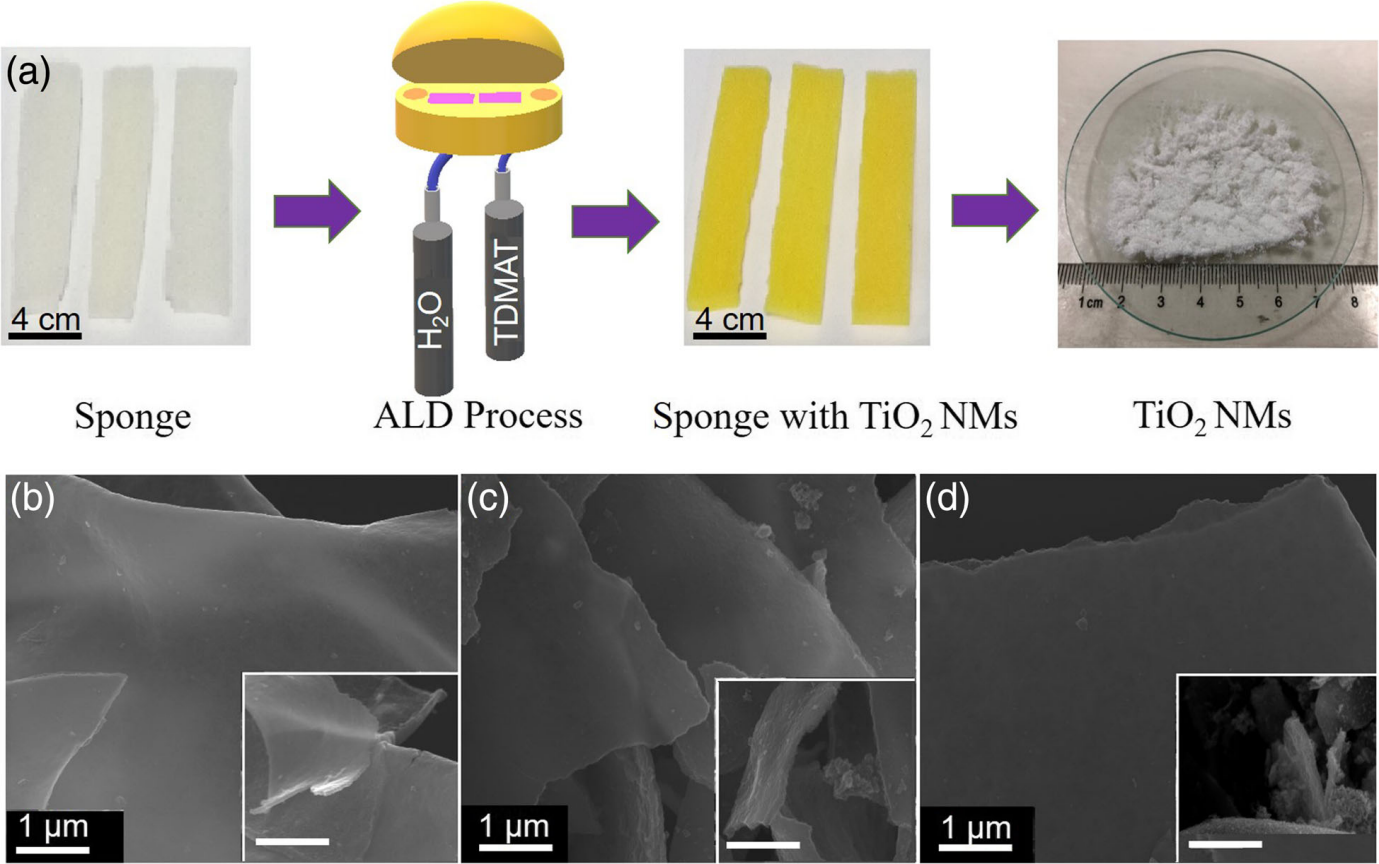
Fabrication process and morphologies of TiO_2_ NMs with different thicknesses. **a** Sketch represented fabrication process of TiO_2_ NMs. **b**–**d** SEM images of TiO_2_ NMs with 100, 200, and 400 ALD cycles, respectively. Scale bars in insets are 1 μm

The preparation of TiO_2_ NMs is shown in Fig. 1a. The TDMAT and H_2_O were used as ALD precursors to deposit TiO_2_ on polyurethane sponge template. The reaction can be described in two half equations as follows: [36]

1 $$ {\displaystyle \begin{array}{l}\mathrm{Ti}{\left(\mathrm{N}{\left({\mathrm{CH}}_3\right)}_2\right)}_4+{\mathrm{TiO}}_2-{\mathrm{OH}}^{\ast}\to \mathrm{NH}{\left({\mathrm{CH}}_3\right)}_2\\ {}+{\mathrm{TiO}}_2-\mathrm{O}-\mathrm{Ti}{{\left(\mathrm{N}{\left({\mathrm{CH}}_3\right)}_2\right)}_3}^{\ast}\end{array}} $$

2 $$ {\displaystyle \begin{array}{l}{\mathrm{TiO}}_2-\mathrm{O}-\mathrm{Ti}{{\left(\mathrm{N}{\left({\mathrm{CH}}_3\right)}_2\right)}_3}^{\ast }+2{\mathrm{H}}_2\mathrm{O}\\ {}\to {\mathrm{TiO}}_2-{\mathrm{TiO}}_2-{\mathrm{OH}}^{\ast }+3\left(\mathrm{N}\mathrm{H}{\left({\mathrm{CH}}_3\right)}_2\right)\end{array}} $$

The total reaction can be written as:

3 $$ \mathrm{Ti}\Big(\mathrm{N}{\left({\mathrm{C}}_2{\mathrm{H}}_6\right)}_4+2{\mathrm{H}}_2\mathrm{O}\to {\mathrm{TiO}}_2+4{\mathrm{H}\mathrm{NC}}_2{\mathrm{H}}_6 $$

**Fig. 2:**
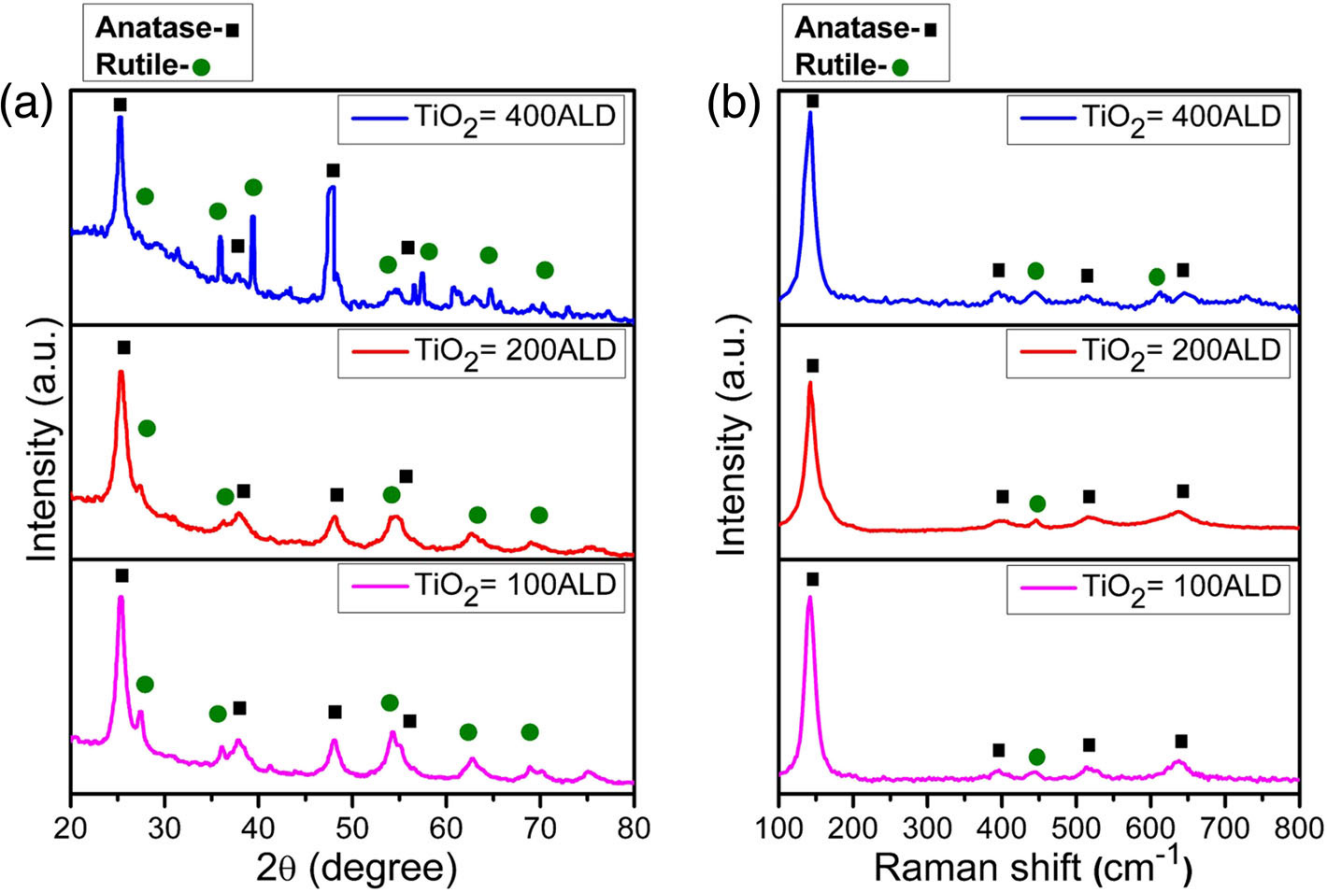
Micro-structural characterizations of TiO_2_ NMs. **a** XRD patterns of TiO_2_ NMs fabricated with 100, 200, and 400 ALD cycles. **b** Raman spectra of TiO_2_ NMs fabricated with 100, 200, and 400 ALD cycles

**Fig. 3:**
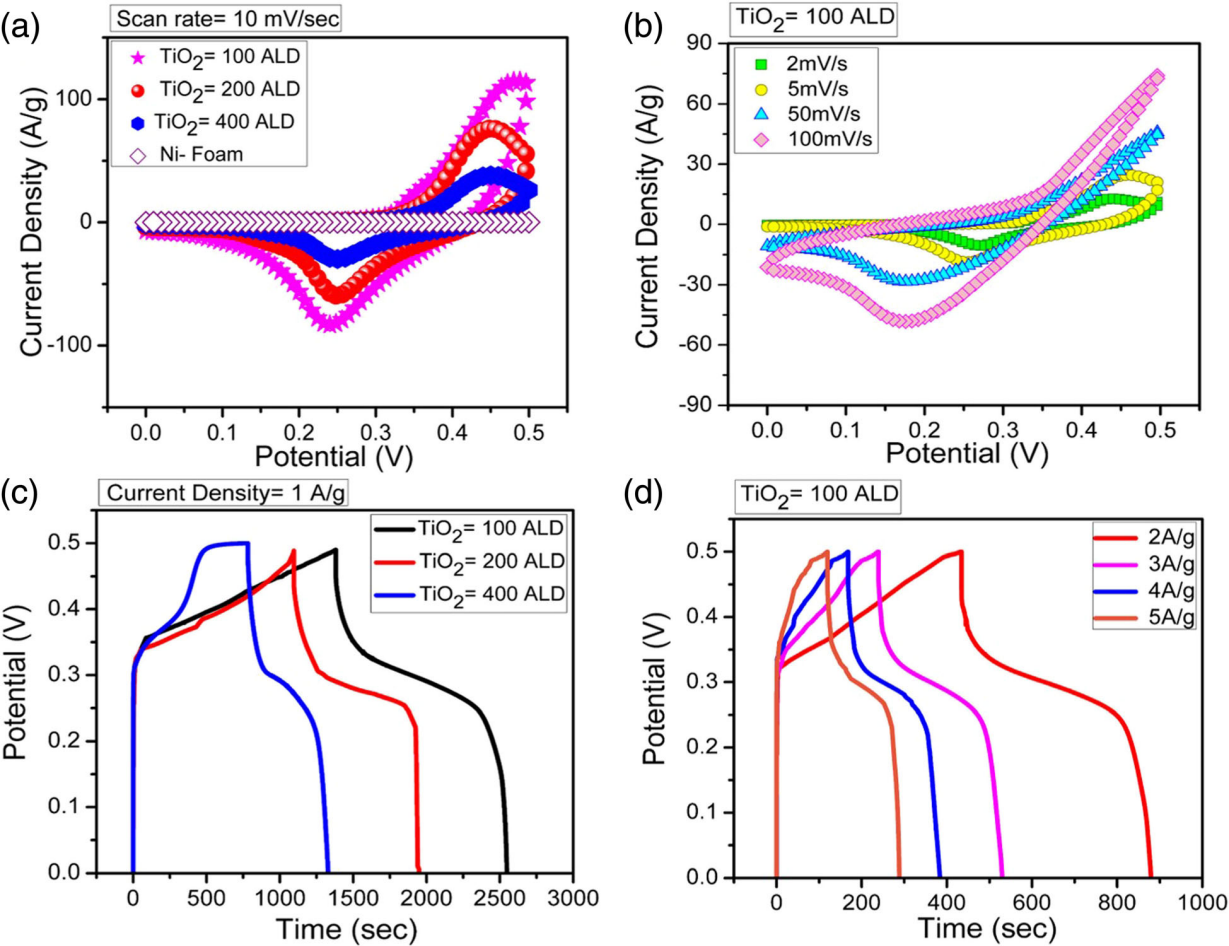
Electrochemical characterization of TiO_2_ NMs supercapacitor. **a** CV curves of pure Ni-foam, electrodes made from TiO_2_ NMs with 100, 200, and 400 ALD cycles. The scan rate is 10 mV/s. **b** CV curves of electrode made from TiO_2_ NMs with 100 ALD cycles, obtained at different scan rates. **c** CP curves of electrode made from TiO_2_ NMs with 100, 200, and 400 ALD cycles. The current density is 1 A/g. **d** CP curve of electrode made from TiO_2_ NMs with 100 ALD cycles, obtained at different current densities

**Fig. 4:**
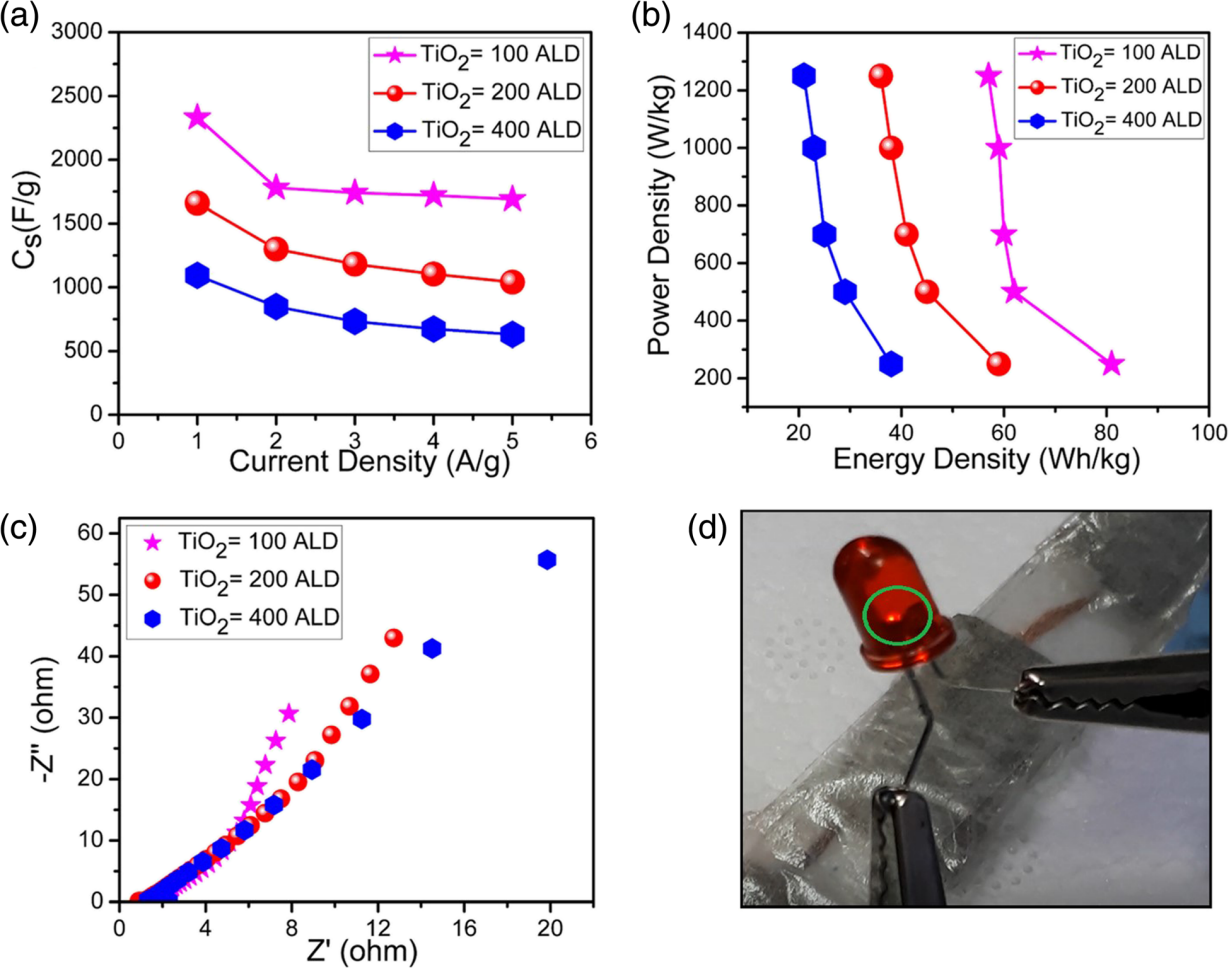
Performance comparison of TiO_2_ NMs electrodes. **a** Specific capacitances of the TiO_2_ NM electrodes at various current densities. **b** Ragone plot of TiO_2_ NMs electrodes with 100, 200, and 400 ALD cycles. **c** Nyquist plot of three TiO_2_ NMs electrodes. **d** A photo showing that two supercapacitors in series can lighten up a red LED

The sponge with TiO_2_ NM coated was then heated to high temperature. During calcination at 500 °C under oxygen atmosphere, the polymer template was converted into CO_2_ and left the 3D porous NM structure behind [34]. Crushing this 3D porous structure led to the fabrication of powder-like structure in white (Fig. 1a). The morphologies of TiO_2_ NMs with 100, 200, and 400 ALD cycles were further observed by SEM and are demonstrated in Fig. 1b–d. We found the lateral sizes of the NMs with different ALD cycles are typically around tens of microns. The thickness of TiO_2_ NMs fabricated in this work was probed by AFM technique and the results are presented in Additional file 1: Figure S1. The average thickness of TiO_2_ NMs with 100, 200, and 400 ALD cycles are ~ 15, 34, and 71 nm, respectively. With the increase of ALD cycles, TiO_2_ NMs is converted into a thicker and stiffer sheet. The corresponding insets in Fig. 1b–d demonstrate that the thickness of NMs is uniform, and some small creases represent the flexibility of TiO_2_ NM especially in the thinner cases. The NMs deposited by ALD can replicate the morphology of the original substrate (i.e., sponge) and therefore some irregular surface structures in the insets of Fig. 1c and d may originate from the template or from the calcination process [37]. Normally, TiO_2_ has three different crystal structures: anatase (tetragonal; space group, *I41/amd*), brookite (orthorhombic; space group, Pcab), and rutile (tetragonal; space group, *P42/mnm*) phases. Here, we carried out detailed characterization to investigate the microstructural properties of TiO_2_ NMs. The crystal structures of the TiO_2_ NMs were investigated by XRD, and the corresponding results are shown in Fig. 2a. The diffraction peaks are indexed to TiO_2_ with anatase and rutile structures (see Additional file 1: Figure S2), indicating the existence of the mixture phase in TiO_2_ NMs calcinated at 500 °C. The co-existence of both phases could be valuable for supercapacitor performance of TiO_2_ NMs [30, 38]. Figure 2b further demonstrates the Raman spectra of corresponding TiO_2_ NMs, which can also be used to identify the phases existed in the NMs. Here, five Raman peaks ascribed to anatase TiO_2_ are located at ~ 142 (E_g_), 393 (B_1g_), 397 (B_1g_), 513 (A_1g_), 515 (A_1g_), and 634 (E_g_) cm^−1^ [39], and they can be observed in all three samples. On the other hand, the 445 cm^−1^ (E_g_) peak is connected with rutile phase and can be seen in all three samples but the Raman peak at 610 cm^−1^ (A_1g_) appears only in TiO_2_ NM with 400 ALD cycles [40]. The emergence of 610 cm^−1^ (A_1g_) peak reflects the microstructural change, which might be caused by the insufficient oxygen for the thick NM during heat treatment in oxygen [41]. This indicates that the increased number of ALD cycles has a remarkable influence on the crystal structure of the TiO_2_ NMs, which can be probed by XRD and Raman spectra shown in Fig. 2. The electronic configuration of the TiO_2_ NMs was also studied by XPS and the results are displayed in Additional file 1: Figure S3. The results prove the existence of Ti^4+^ in all NMs and a small shift of the peaks may be ascribed to the change in crystal structure as mentioned above. In order to study the electrochemical performance of the TiO_2_ NMs, three-electrode electrochemical system including a reference electrode, counter electrode, and a working electrode was operated. Here, Ag/AgCl was served as a reference electrode to control the potential difference and Pt counter electrode was engaged as an electron source to transit current towards TiO_2_ NMs working electrode in the presence of aqueous electrolyte (1 M KOH solution). It is worth noting that the functional voltage of supercapacitor depends on the electrolyte, and aqueous electrolyte with well electronic conductivity and high dielectric constant may be helpful in attaining higher capacitance [42]. The acquired CV and CP curves of electrodes made from TiO_2_ NMs with 100, 200, and 400 ALD cycles are displayed in Fig. 3a, b and Additional file 1: Figure S4. One can see that in Fig. 3a, all CV curves of three electrodes made from TiO_2_ NMs with different thicknesses exhibit redox peaks. The CV curve of pure nickel foam is also plotted for comparison, and no obvious peak can be observed. Generally, the appearance of redox peaks can be associated to cation interactions on the surface of the TiO_2_ NMs, and the interaction can be expressed as: [43]

$$ {\left({\mathrm{TiO}}_2\right)}_{\mathrm{surface}}+{\mathrm{M}}^{+}+{e}^{-}\leftrightarrow {\left({\mathrm{TiO}}_2{{}^{-}\mathrm{M}}^{+}\right)}_{\mathrm{surface}} $$

where M^+^ could be H_3_O^+^or K^+^ in the electrolyte. The change between different oxidation states of Ti ion suggests its potential as redox electrode material. In response of fast surface Farad reaction, the CV curves of TiO_2_ NMs exhibit larger areas compared with that of pure Ni-foam, implying the higher specific capacitance value of TiO_2_ NMs. Specifically, one can see the area of the CV curves decreases with the ALD cycles, suggesting a decrease of capacitance in the case of thicker NMs, as will be further proved in following CP results. A reduction peak at ~ 0.2 V can be clearly observed in all the electrodes and is associated with intraband gap localized states [44, 45]. In addition, we also measured CV curves of electrode made from TiO_2_ NMs with 100 ALD at different scan rates, and the results are shown in Fig. 3b. A redox peak shifting behavior (from higher to lower potential) is connected with the change in intercalation/deintercalation of M^+^ ions and synergetic effect [46, 47]. Briefly, limited diffusion and charge transfer rate at a higher scan rate lead to corresponding shift [48, 49]. In order to further illustrate the charging/discharging behavior, the galvanostatic charge/discharge curves of TiO_2_ NMs electrodes at different current densities within a potential range of 0–0.5 V are shown in Fig. 3c, d and Additional file 1: Figure S4. The nonlinear curves of CP represent the pseudocapacitor function, which is consistent with the CV curves, and represent the Faradaic behavior. It should be noted that the discharge time of TiO_2_ NMs electrode with 100 ALD cycles is notably prolonged compared with TiO_2_ NMs electrodes with 200 and 400 ALD cycles, indicating the largest specific capacitance value. However, ultra-thin NMs electrode exhibit high gravimetric specific activity but cannot afford large current due to the limited number of active sites [50]. The extended charging/discharging times of TiO_2_ NMs electrodes with 100, 200, and 400 ALD cycles at current density of 1 A/g means that reduction/oxidation reactions take place (mainly on surfaces of NMs) during the process, which is the property of pseudocapacitor [51]. Figure 4 (a) shows the specific capacitances of electrodes made from TiO_2_ NMs with 100, 200, and 400 ALD cycles at different current densities ranging from 1 to 5 A/g. Specific capacitances of 2332, 1780, 1740, 1720, and 1690 F/g are obtained from TiO_2_ NMs with 100 ALD cycles, 1660, 1300, 1182, 1104, and 1040 F/g from TiO_2_ NMs with 200 ALD, and 1094, 848, 732, 672, and 630 F/g from TiO_2_ NMs with 400 ALD cycles. In previous literature, Yang et al. [43] prepared the TiO_2_/N-doped graphene composite structure with a capacitance of 385.2 F/g at 1 A/g and 320.1 F/g at 10 A/g. Zhi et al. [52] reported a specific capacitance of 216 F/g for TiO_2_ nanobelts with nitrogen doping. Di et al. [53] fabricated TiO_2_ nanotubes decorated with MnO_2_ nanoparticles and a specific capacitance of 299 F/g at a current density of 0.5 A/g was obtained. Obviously, the capacitance of the electrode made from current TiO_2_ NMs is much higher. Moreover, the energy and power density relation of the three electrodes are shown in Fig. 4b and Additional file 1: Table S1. Energy density is the capacity of energy storage devices and power density is their ability to deliver it, and both are the key parameters used to evaluate the electrochemical performance of supercapacitors. Vividly, when current density increases from 1 to 5 A/g, TiO_2_ NMs electrode with 100 ALD cycles possesses a high energy density of 81–57 Wh/kg compared to 59–36 Wh/kg of TiO_2_ NMs electrode with 200 ALD cycles and 38–21 Wh/kg of TiO_2_ electrode NMs with 400 ALD cycles, while the power density increases from 250 to 1250 W/kg (Fig. 4b). The high performance might be due to the mixture of anatase and rutile phases (Fig. 2) as this leads to surface passivation and increased ion transportation [54–56]. In addition, the enlarged surface area of the TiO_2_ NMs and interconnectivity among the NMs also cause the enhancement in ions transportation. On the other hand, we believe that the decrease in electrochemical performance with the increasing ALD cycles is mainly due to the decreased NM/electrolyte interface area if the masses of the active materials are the same. Moreover, the TiO_2_ NMs with more ALD cycles (i.e., thickness) is stiffer and flat (see Fig. 1), and therefore, the overlap between the NMs is obvious. This may limit the surface access for electrolyte ions, resulting in dead volume, high resistance, and reduced capacitance [57]. In addition, with the increase of current densities, the diffusion rate of electrolyte might not be enough to satisfy the electrochemical reaction of electrode material, and therefore, a decrease of capacitance with current density can be observed in Fig. 4a [39, 40]. In order to further reveal the electrochemical properties of the current TiO_2_ NMs electrodes, EIS characterizations was carried out because EIS can provide the information about electrode-electrolyte and electrode internal resistance [58]. Figure 4c demonstrates the EIS results of all three electrodes, and the horizontal intercept indicates the internal resistance of pseudocapacitor. It is clearly observed that TiO_2_ NMs electrode with 400 ALD cycles possesses high internal resistance as compared to TiO_2_ NMs electrodes with 200 and 100 ALD cycles. We consider that the increased resistance of TiO_2_ NMs electrode with 400 ALD cycles is mostly by reason of increased NM thickness since the TiO_2_ has relatively large resistivity [39, 48]. The TiO_2_ NMs with 100 ALD cycles exhibits the lowest internal resistance compared with others because the large surface area allows the better ions passage [59] and flexibility of thin NM improves the interlayer connection with decreased resistivity. All these results demonstrate that thin TiO_2_ NMs with high electroactivity are promising electrode materials for high-performance pseudocapacitor. In order demonstrate the potential application of TiO_2_ NMs supercapacitor, four electrodes made from TiO_2_ NMs with 100 ALD cycles were assembled into two symmetrical supercapacitors, i.e., each supercapacitor consisted of two electrodes of TiO_2_ NMs with 100 ALD cycles. The two supercapacitors were connected in series and then charged at 5 A/g current density to 0.5 V. Afterwards, they were used to light up a red LED (light-emitting diode) with working voltage of ~ 1.5 V and the LED emitted light for ~ 1 min (see Fig. 4d and Additional file 2: Video S1). The cycle stability of the electrode made from TiO_2_ NMs with 100 ALD cycles was also studied and the results are shown in Additional file 1: Figure S5. A capacitance retention of 80.98% is observed after cycling at 5 A/g for 40 charge/discharge cycles, suggesting a less interaction of electrolyte ions with electrode surface after repeated cycles. We believe that the performance of the NMs electrode might be further promoted if the conductivity of the NMs is increased. With the help of the ALD technique, the conductivity of the NMs can be increased by fabricating multi-layered NMs where materials with high conductivity are incorporated. More works are currently in progress.

## Conclusion

In summary, we have fabricated TiO_2_ NMs for electrodes of supercapacitor, and the electrochemical performance of the NMs was studied in detail. The TiO_2_ NM electrode demonstrates increased capacitance with deceased NM thickness. At a current density of 1 A/g, the specific capacitance of 2332 F/g is obtained for TiO_2_ NM with 100 ALD cycles, and the corresponding energy density is calculated to be 81 Wh/kg. The enhancement of the performance is mainly attributed to the fabrication strategy and the ultra-thin feature of NMs, because the large surface area and short diffusion path of NMs facilitate ion transport through electrode/electrolyte interface. The interconnectivity among the NMs also remarkably enhances the ion transportation in the electrode. We also demonstrate that two supercapacitors connected in series can power a LED, suggesting the application potential of TiO_2_ NMs supercapacitor. The current facile design opens the way to build NMs electrodes for next-generation wearable energy storage devices at low-cost. However, for practical applications of NM-based structures in future supercapacitors, further studies are required.

## Additional files

Additional file 1: **Figure S1.** Surface morphologies of ALD synthesized TiO_2_ NMs with different ALD cycles: (a) 100 ALD cycles. (b) 200 ALD cycles. (c) 400 ALD cycles. **Figure S2.** Crystal structures of TiO_2_. (a) Diagram showing the arrangement of atoms in anatase and rutile phases of TiO_2_. (b) Standard XRD patterns of anatase TiO_2_ (JPCDS # 21–1272) and rutile TiO_2_ (JPCDS # 03–1122). **Figure S3.** XPS spectra of TiO_2_ NMs with 100, 200, and 400 ALD cycles. To calibrate, C 1s peak is used as reference peak at binding energy of 284.6 eV. High-resolution XPS spectra of (a) Ti 2p and (b) O 1 s. The peaks at ∼464.9 and ∼459 eV is assigned to Ti^4+^ 2p1/2 and Ti^4+^ 2p_3/2_ respectively. The peak at 529 eV is assigned to O 1 s. **Figure S4.** Electrochemical characterization of TiO_2_ NMs. (a) and (c) CV curves of TiO_2_ NMs with 200 and 400 ALD cycles at different scan rates. (b) and (d) CP curves of TiO_2_ NMs with 200 and 400 ALD cycles at different current densities. **Figure S5.** Cycle performance of electrode made from TiO_2_ NMs with 100 ALD cycles. **Table S1.** Comparison of specific capacitance and energy density of electrodes made from TiO_2_ NMs with different ALD cycles. (DOCX 2102 kb)

Additional file 2: **Video S1.** Two supercapacitors in series can lighten up a red LED. (MP4 1136 kb)

## Abbreviations

AFM: Atomic force microscopy
ALD: Atomic layer deposition
CP: Chronopotentiometry
CV: Cyclic voltammetry
DI: De-ionized water
EIS: Electrochemical impedance spectroscopy
LED: Light-emitting diode
NMs: Nanomembranes
PTFE: Polytetrafluoroethylene
SEM: Scanning electron microscopy
TDMAT: Tetrakis dimethylamide titanium
XPS: X-ray photoelectron spectroscopy
XRD: X-ray diffraction spectrometer
