# Supplementary material for: TiO2 Nanomembranes Fabricated by Atomic Layer Deposition for Supercapacitor Electrode with Enhanced Capacitance
Source: Nanoscale Res Lett. 2019 Mar 13;14:92. doi: 10.1186/s11671-019-2912-3 (PMC6419636; doi:10.1186/s11671-019-2912-3)
Supplement: Supplementary file 1 — Figure S1. Surface morphologies of ALD synthesized TiO2 NMs with different ALD cycles: (a) 100 ALD cycles. (b) 200 ALD cycles. (c) 400 ALD cycles. Figure S2. Crystal structures of TiO2. (a) Diagram showing the arrangement of atoms in anatase and rutile phases of TiO2. (b) Standard XRD patterns of anatase TiO2 (JPCDS # 21–1272) and rutile TiO2 (JPCDS # 03–1122). Figure S3. XPS spectra of TiO2 NMs with 100, 200, and 400 ALD cycles. To calibrate, C 1s peak is used as reference peak at binding energy of 284.6 eV. High-resolution XPS spectra of (a) Ti 2p and (b) O 1 s. The peaks at ∼464.9 and ∼459 eV is assigned to Ti4+ 2p1/2 and Ti4+ 2p3/2 respectively. The peak at 529 eV is assigned to O 1 s. Figure S4. Electrochemical characterization of TiO2 NMs. (a) and (c) CV curves of TiO2 NMs with 200 and 400 ALD cycles at different scan rates. (b) and (d) CP curves of TiO2 NMs with 200 and 400 ALD cycles at different current densities. Figure S5. Cycle performance of electrode made from TiO2 NMs with 100 ALD cycles. Table S1. Comparison of specific capacitance and energy density of electrodes made from TiO2 NMs with different ALD cycles. (DOCX 2102 kb) [file 11671_2019_2912_MOESM1_ESM.docx]

**Additional file 1**

**TiO_2_ nanomembranes fabricated by atomic layer deposition for supercapacitor electrode with enhanced capacitance**

Farah Naeem^1, 2^, Sumayyah Naeem^1, 2^, Yuting Zhao^1^, Dingrun Wang^1^, Jing Zhang^3^, YongFeng Mei^1^, and Gaoshan Huang^1^

^1^Department of Materials Science, Fudan University, 220 Handan Road, Shanghai 200433, P.R. China.

^2^ State Key Laboratory for Modification of Chemical Fibers and Polymer Material Science and Engineering, Donghua University, Shanghai 201620, P. R. China.

^3^ College of Science, Donghua University, Shanghai 201620, P. R. China.


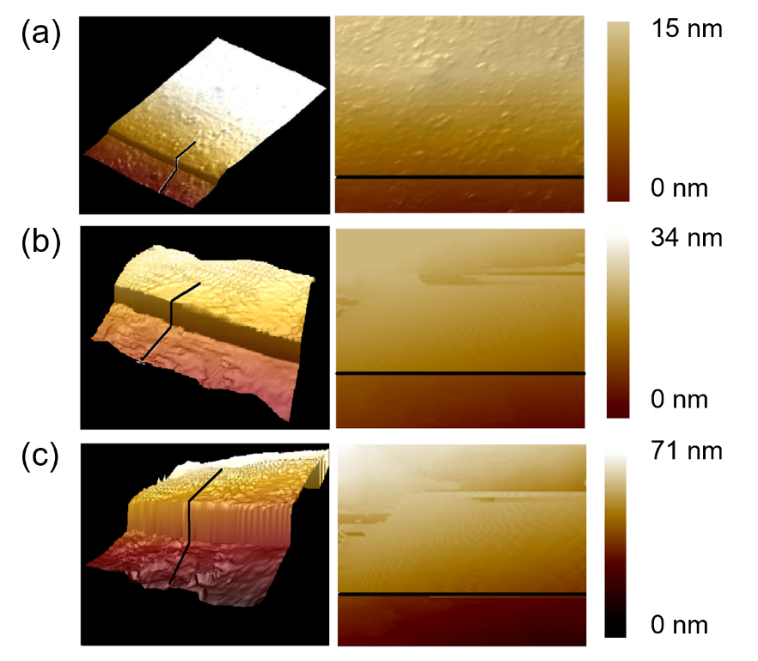


**Figure S1** Surface morphologies of ALD synthesized TiO_2_ NMs with different ALD cycles: (a) 100 ALD cycles. (b) 200 ALD cycles. (c) 400 ALD cycles.


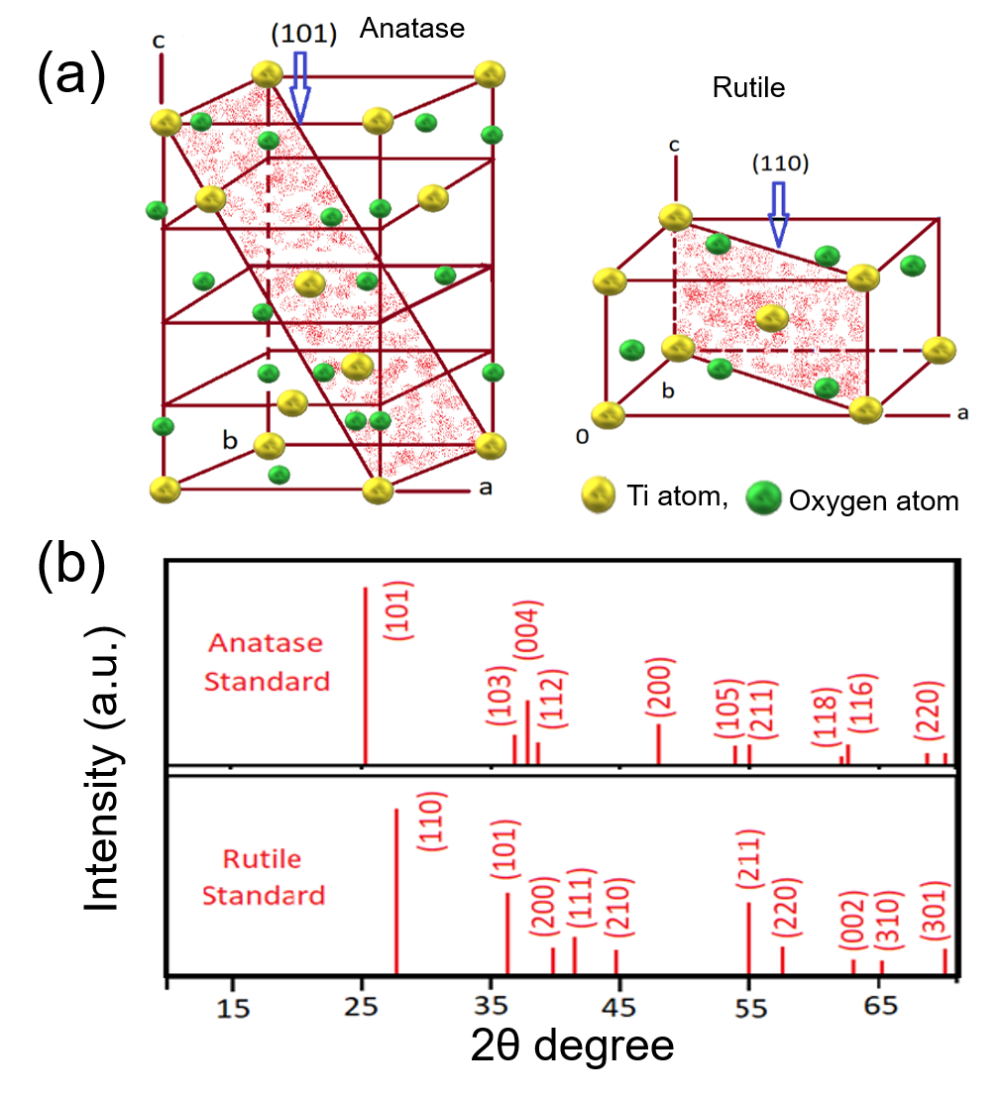


**Figure S2** Crystal structures of TiO_2_. (a) Diagram showing the arrangement of atoms in anatase and rutile phases of TiO_2_. (b) Standard XRD patterns of anatase TiO_2_ (JPCDS # 21- 1272) and rutile TiO_2_ (JPCDS # 03- 1122).

**
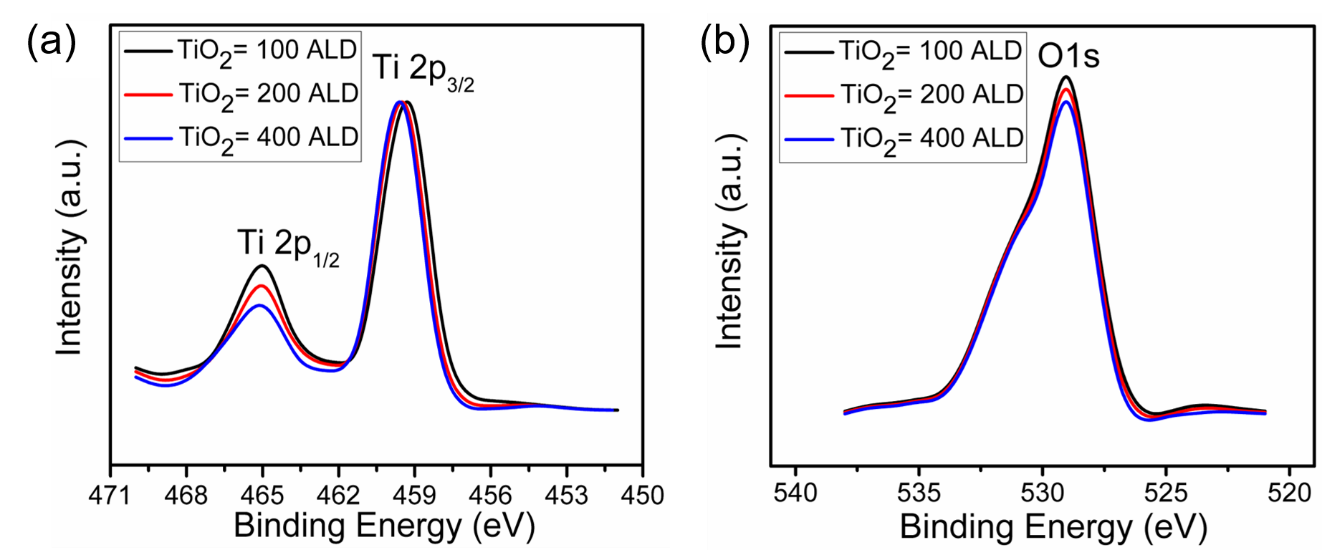
**

**Figure S3** XPS spectra of TiO_2_ NMs with 100, 200, and 400 ALD cycles. To calibrate, C 1s peak is used as reference peak at binding energy of 284.6 eV. High resolution XPS spectra of (a) Ti 2p and (b) O 1s. The peaks at ∼464.9 and ∼459 eV is assigned to Ti^4+^ 2p1/2 and Ti^4+^ 2p_3/2_ respectively. The peak at 529 eV is assigned to O 1s.


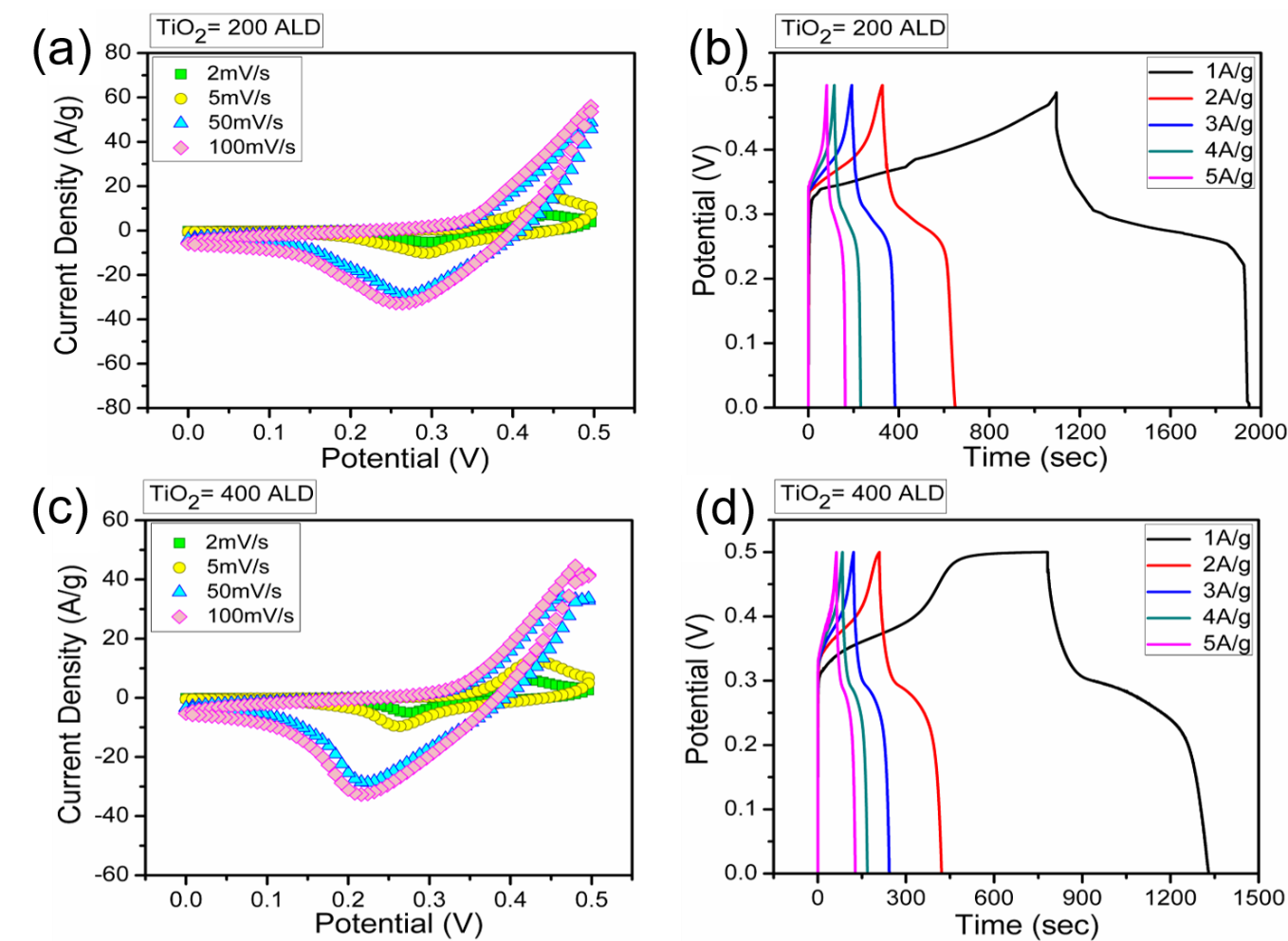


**Figure S4** Electrochemical characterization of TiO_2_ NMs. (a) and (c) CV curves of TiO_2_ NMs with 200 and 400 ALD cycles at different scan rates. (b) and (d) CP curves of TiO_2_ NMs with 200 and 400 ALD cycles at different current densities.


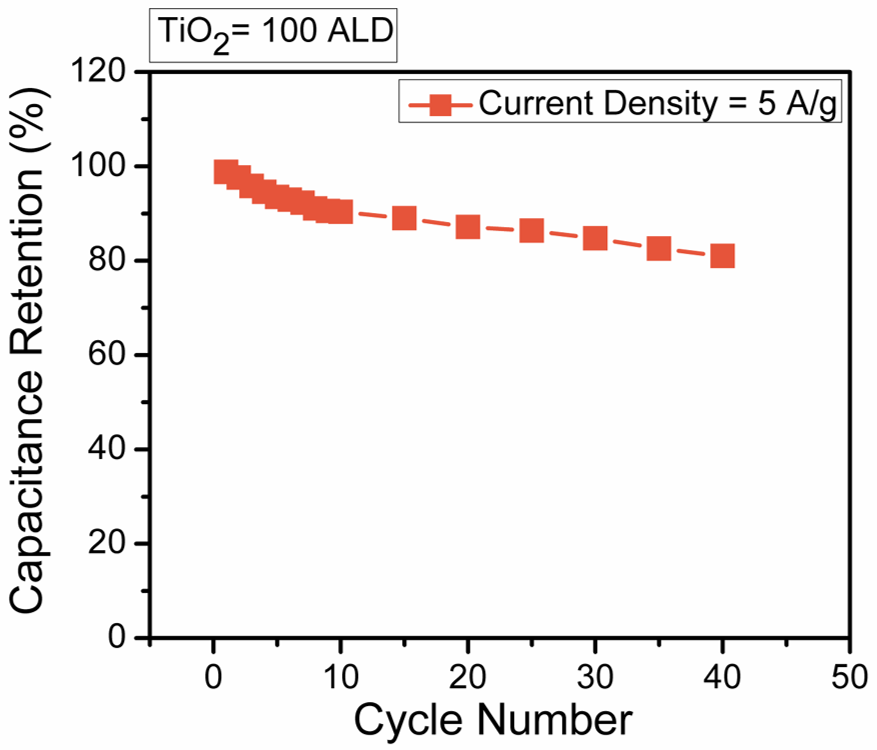


**Figure S5** Cycle performance of electrode made from TiO_2_ NMs with 100 ALD cycles.

**Table S1** Comparison of specific capacitance and energy density of electrodes made from TiO_2_ NMs with different ALD cycles.

| **TiO_2_ NM** | **Specific Capacitance (F/g)**  **at 1 A/g** | **Energy Density (Wh/Kg)** |
| --- | --- | --- |
| 100 ALD cycles | 2332 | 81 |
| 200 ALD cycles | 1660 | 59 |
| 400 ALD cycles | 1094 | 38 |

**Electrochemical performance calculation method**

**Calculation of gravimetric capacitance, energy**

The specific capacitance (C) is calculated from the CP data,

$C_{s}=J\frac{\Delta t}{\Delta v}(F/g)$ (s1)

where, J is current density (A/g), ∆t is discharge time (sec), ∆V is voltage window (V).

The energy density (E) was calculated according to the following equation:

$E=\frac{C*\Delta V^{2}*1000}{2*3600}(Wh/kg)$ (s2)

where, C is specific capacitance (F/g), ∆V is voltage window (V).

The power density (P) was calculated according to the following equations:

$P=\frac{E*3600}{\Delta t}(W/kg)$ (s3)

where, E is energy density (Wh/kg), ∆t is discharge time (sec).
